# Cerebral Infarction After Switching From Roxadustat to Daprodustat in a Patient With Renal Anemia

**DOI:** 10.7759/cureus.63942

**Published:** 2024-07-06

**Authors:** Kohzo Takebayashi, Mototaka Yamauchi, Kenji Hara, Takafumi Tsuchiya, Koshi Hashimoto

**Affiliations:** 1 Department of Diabetes, Endocrinology and Hematology, Dokkyo Medical University Saitama Medical Center, Koshigaya, JPN

**Keywords:** daprodustat, diabetes type 2, acute cerebral infarction, central hypothyroidism, roxadustat

## Abstract

Renal anemia is generally caused by a decrease in the production of erythropoietin in kidney due to renal dysfunction, and this may be associated with the increase in mortality and cardiovascular events in addition to subjective symptoms such as fatigue and wobbliness.

We report a case of an 87-year-old man with type 2 diabetes, hypertension, and dyslipidemia who had received roxadustat (a hypoxia-inducible factor (HIF) prolyl hydroxylase (PH) inhibitor) for renal anemia due to diabetic nephropathy and in whom roxadustat was switched to daprodustat (another HIF-PH inhibitor) due to the onset of central hypothyroidism. About three weeks after this change, the patient developed acute asymptomatic cerebral infarction with an elevation of hemoglobin (Hb). It is unclear if the change to daprodustat was involved in the onset of cerebral infarction. However, this case suggests that particular caution should be paid to unexpected acute elevation of Hb after a change from one HIF-PH inhibitor to another, especially in a patient at high risk for cardiovascular events.

## Introduction

Hypoxia-inducible factor (HIF) prolyl hydroxylase (PH) inhibitors are now widely used clinically for the treatment of renal anemia. These drugs can be orally administered, and this may be an advantage compared with erythropoiesis-stimulating agents (ESAs), which are drugs for renal anemia that are administered intravenously or subcutaneously. In Japan, five HIF-PH inhibitors are currently clinically available. Roxadustat, which was first approved for clinical use in Japan, has the unique advantage of requiring administration only three times a week, compared with other HIF-PH inhibitors given once daily. However, a possible association of roxadustat with an important adverse event of central hypothyroidism (CeH) has recently become clear [1-6]. The molecular structure of roxadustat is similar to 3,3′,5-triiodothyronine (T3), and therefore, may act as a selective thyroid hormone receptor (THR)-β agonist in the pituitary gland with higher affinity for THR-β compared with T3 [7]. This may result in CeH due to the so-called negative feedback effect for the production and secretion of thyroid-stimulating hormone (TSH) in the pituitary gland. In fact, in the amended Japanese pharmaceutical package insert of roxadustat, CeH has been added as a possible adverse event, based on the accumulation of clinical reports.

In a case with CeH due to roxadustat, a change from roxadustat to another HIF-PH inhibitor may also require a warning due to the unexpected acute elevation of hemoglobin (Hb) in blood, especially in elderly patients with cardiovascular risk factors. However, there is incomplete information on the safety and efficacy of a change from one HIF-PH inhibitor to another. Here, to raise awareness of this issue, we report a case of an elderly patient with type 2 diabetes, hypertension, and dyslipidemia who received roxadustat for renal anemia due to diabetic nephropathy. Roxadustat was switched to daprodustat (another HIF-PH inhibitor) at the minimum dose used clinically (2 mg daily) due to the onset of CeH, and acute asymptomatic cerebral infarction developed about three weeks after the change of drugs.

## Case presentation

An 87-year-old man with type 2 diabetes, hypertension, and dyslipidemia for over 20 years had been suspected to have developed diabetic nephropathy based on elevated serum creatinine (Cr) and proteinuria (+) in 2007. During this period of over 20 years, his hemoglobin (Hb) A1c level as a measure of glycemic control had been maintained at approximately 7% by insulin therapy, with occasional transient exacerbation (i.e., elevation of HbA1c). Regarding renal function, Cr had gradually increased, and laboratory data in 2011 showed Cr 1.3 mg/dL, estimated glomerular filtration rate (eGFR) 42.2 mL/min/1.73 m^2^, and urinary albumin excretion (UAE) 66.9 mg/g Cr. These data are consistent with stage 2 diabetic nephropathy, as defined by the Japan Diabetes Society. At this time point, the Hb level was 11.6 g/dL. This level gradually decreased with the progression of probable diabetic nephropathy and was 9 g/dL in 2019.

In 2021, Cr was 1.87 mg/dL, eGFR 27.4 mL/min/1.73 m^2^, Hb 9.3 g/dL, Fe 163 μg/dL, total iron binding capacity (TIBC) 456 μg/dL, and ferritin 15.4 ng/mL. Therefore, roxadustat 50 mg three times weekly was initiated as a treatment for renal anemia due to the progression of diabetic nephropathy. After five months, Hb had improved to 12.4 g/dL, but the patient had noted occasional mild finger tremors and palpitation. Thus, for the differential diagnosis of possible hyperthyroidism, a thyroid function blood test was performed, and the results showed FT4 1.7 μg/dL, FT3 2.3 pg/mL, and TSH 0.03 μU/mL, suggesting an upper limit of FT4 and the decrease of TSH. However, elevated levels of thyroglobulin antibody (TgAb) (784 IU/mL) and thyroid peroxidase antibody (TPOAb) (152 IU/mL) were also noted, while TSH receptor antibody (TRAb) was negative. The normal TRAb and elevated TgAb and TPOAb levels suggested transient subclinical thyrotoxicosis due to painless thyroiditis based on chronic thyroiditis rather than Graves’ disease. In fact, thyroid function improved in follow-up over four months, with FT4 1.08 μg/dL, FT3 1.88 pg/mL, and TSH 2.50 μU/mL. On the same day, sodium ferrous citrate 100 mg daily was initiated because of relatively low Fe (54 μg/dL) and ferritin (10.0 ng/mL) and high TIBC (445 μg/dL).

Administration of roxadustat was repeatedly stopped and resumed based on the Hb level (target level: approximately 11 g/dL). In 2023, Hb was 7.9 g/dL, mean corpuscular volume (MCV) 102.5 fl, Fe 123 μg/dL, TIBC 241 ug/dL, ferritin 372 ng/mL, Cr 3.19 mg/dL, and eGFR 15.1 mL/min/1.73 m^2^, suggesting greater exacerbation of renal anemia due to diabetic nephropathy, and thus, roxadustat was restarted for the first time in two months. Thyroid function data on the same day were similar, with FT4 1.23 μg/dL, FT3 1.63 pg/mL, and TSH 3.57 uU/mL, in the normal range. However, after six months, although Hb had improved to 11.6 g/dL, the data for FT4 0.79 μg/dL, FT3 2.26 pg/mL, and TSH 0.334 μU/mL suggested mild CeH.

**Figure 1:**
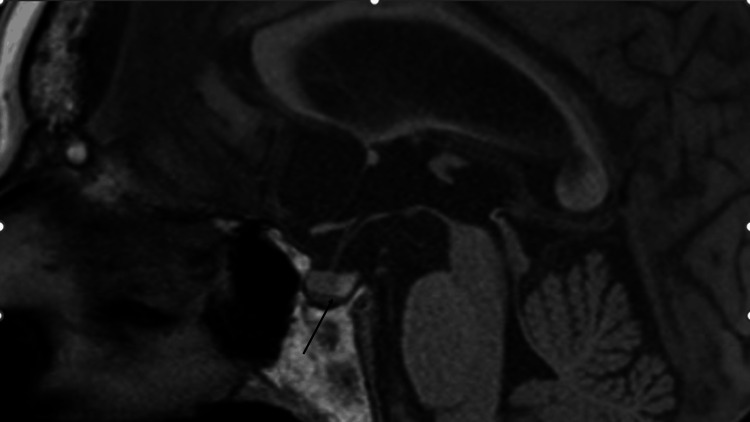
T1-weighted image in pituitary magnetic resonance imaging (MRI). Pituitary MRI shows normal findings. Arrow shows normal findings of the pituitary.

**Figure 2:**
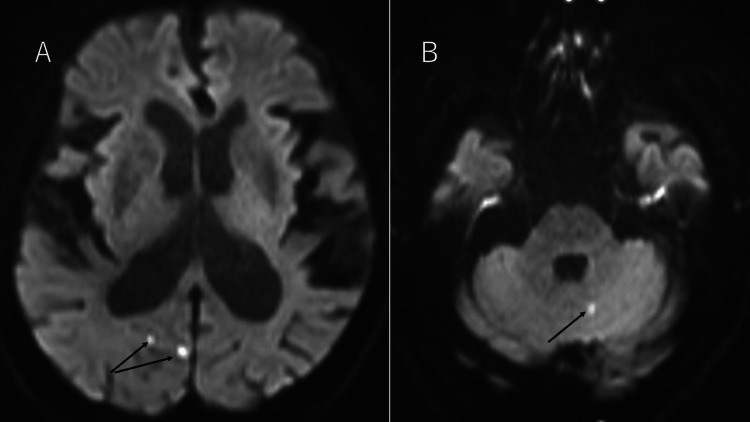
(A) Diffusion-weighted images in brain magnetic resonance imaging (MRI). (B) Diffusion-weighted images in MRI in the cerebellar hemisphere. In (A) and (B), arrows show the portion of acute cerebral infarction.

A blood test after four months showed a similar finding of CeH (FT4 0.88 μg/dL, FT3 1.56 pg/mL, and TSH 0.489 uU/mL), and roxadustat was changed to daprodustat 2 mg daily because of the possible induction of CeH by roxadustat. The Hb level on the same day was 10.6 g/dL. After three weeks, the patient revisited our hospital for brain and pituitary magnetic resonance imaging (MRI). The pituitary MRI showed normal findings (Figure 1), but the brain MRI incidentally showed acute infarction in the right cerebral subcortex of the occipital lobe and left cerebellar hemisphere (Figures 2A, 2B).

Laboratory data on the same day showed FT4 0.86 μg/dL and TSH 0.041 μU/mL, suggesting the progression of CeH and Hb 12.7 μg/dL.

A diagnosis of asymptomatic cerebral infarction was made because the patient’s level of consciousness was completely clear, and there were no apparent neurological findings. He was hospitalized in the neurology department of our hospital on the next day for follow-up and related tests. Laboratory data at the time of hospitalization are shown in Table 1.

**Table 1:** Laboratory data on hospitalization. WBC: white blood cell count, RBC: red blood cell count, Hb: hemoglobin, Ht: hematocrit, MCV: mean corpuscular volume, MCH: mean corpuscular hemoglobin, Plt: platelet, TP: total protein, Alb albumin, AST: aspartate transaminase, ALT: alanine transaminase, ALP: alkaline phosphatase, LDH: lactate dehydrogenase, GGT: gamma-glutamyl transpeptidase, T-Bil: total bilirubin, D-Bil: direct bilirubin, Na: sodium, K: potassium, Cl: chlorine, BUN: urea nitrogen, Cr: creatinine, eGFR: estimated glomerular filtration rate, UA: uric acid, Fe: iron, TIBC: total iron binding capacity, FPG: fasting plasma glucose, HbA1c: hemoglobin A1c, UAE: urinary albumin excretion, PT: prothrombin time, APTT: activated partial thromboplastin time, FDP: fibrin/fibrinogen degradation products, FT4: free thyroxine, TSH: thyroid-stimulating hormone, FT3: free triiodothyronine, TgAb: thyroglobulin antibody, TPOAb: thyroid peroxidase antibody, ACTH: adrenocorticotropic hormone, GH: growth hormone, PRL: prolactin, LH: luteinizing hormone, FSH: follicle stimulating hormone.

Test (unit)	Observed value	Reference value
Blood test		
WBC (/μL)	5300	3300-8600
RBC (×10^4^/μL)	356	435-555
Hb (g/dL)	12.7	13.7-16.8
MCV (fl)	107	83.6-98.2
MCH (pg)	35.7	27.5-33.2
Plt (×10^4^/μL)	14.2	15.8-34.8
Chemistry et al.		
TP (g/dL)	7.5	6.6-8.1
Alb (g/dL)	3.63	4.1-5.1
AST (U/L)	31	13-30
ALT (U/L)	23	10-42
LDH (U/L)	386	124-222
ALP (U/L)	110	38-113
GGT (U/L)	19	13-64
T-Bil (mg/dL)	1.53	0.4-1.53
D-Bil (mg/dL)	1.32	0.1-0.3
Na (mEq/L)	142	138-145
K (mEq/L)	5	3.6-4.8
Cl (mEq/L)	108	101-108
BUN (mg/dL)	44	8-2.0
Cr (mg/dL)	2.83	0.65-1.07
eGFR (mL/min/1.73 m^2^)	17.2	No reference value
UA (mg/dL)	4	3.7-7.8
Fe (μg/mL)	102	40-188
Ferritin (ng/mL)	344.5	45.9-322.3
TIBC (μg/mL)	257	258-411
Vitamin B12 (pg/mL)	196	180-914
Folic acid (ng/mL)	4.5	>4
Erythropoietin (mIU/mL)	6.6	4.2-23.7
Data related to diabetes		
FPG (mg/dL)	97	73-109
HbA1c (%)	6.8	4.9-6
UAE (mg/g Cr)	2234.9	<30
Coagulation test		
PT (%)	120.3	80-120
APTT (sec)	26.7	No reference value
Fibrinogen (mg/dl)	283	200-400
D-dimer (μg/mL)	5.6	0-1
FDP (μg/mL)	7.8	<5.0
Data related to thyroid		
FT4 (μg/dL)	0.86	0.90-1.70
TSH (μU/mL)	0.041	0.5-5
One week after hospitalization		
FT4 (μg/dL)	0.79	0.90-1.70
FT3 (ng/mL)	1.51	2.3-4.0
TSH (μU/mL)	1.19	0.5-5
TgAb (IU/mL)	1100	0-26
TPOAb (IU/mL)	126	0-16
Other hormones		
ACTH (pg/mL)	48	7.2-63.3
GH (ng/mL)	0.72	<2.47
PRL (ng/mL)	20.5	4.29-13.69
LH (mIU/mL)	70.4	No reference value
FSH (mIU/mL)	107	No reference value
Cortisol (μg/dL)	18.3	7.07-63.3

After admission, transthoracic echocardiography and an ultrasound examination of the lower limb veins showed patent foramen ovale (PFO) and stable venous thrombosis in the lower limb, respectively. Therefore, although the association between brain infarction and venous thrombosis was not clear, we considered that the patient had a high risk of paradoxical cerebral embolism. Thus, the temporal administration of warfarin was started. Furthermore, to decrease the risk of recurrence of cerebral infarction due to elevated Hb, daprodustat was temporarily suspended. Laboratory data at one week after admission were FT4 0.79 μg/dL, FT3 1.51 pg/mL, TSH 1.19 μU/mL, and Hb 11.7 g/dL. There were no problems in clinical findings during hospitalization and the patient was discharged after about three weeks of hospitalization.

## Discussion

In this case, the first thyroid function blood test was performed after roxadustat had been started, and this test indicated a decrease of TSH with FT4 at the upper limit, negative TRAb, and positive TgAb and TPOAb. Therefore, we considered that the patient had developed painless thyroiditis based on suspected chronic thyroiditis. Thyroid function improved with follow-up without medication after four months. Thus, it is unclear if continuous roxadustat influenced the transient thyroid dysfunction. There is no clear evidence that roxadustat can cause or promote destructive thyroiditis, such as painless thyroiditis, and this is an interesting issue for further investigation.

About six months after restarting roxadustat, decreases in FT4, FT3, and TSH were noted, suggesting the onset of CeH. A similar finding was obtained four months later. Thus, we concluded that this was due to CeH as an adverse event of roxadustat, and therefore, roxadustat 50 mg three times weekly was switched to daprodustat (another HIF-PH inhibitor) 2 mg daily, which is the minimum dose of this drug used clinically. Interestingly, TSH further decreased to 0.041 μU/mL three weeks after the change to daprodustat, but that was probably still due to the influence of roxadustat rather than daprodustat because the suppression of TSH continued for a few weeks after discontinuation of roxadustat [3]. In a case described in the amended package insert of roxadustat, TSH was also decreased at three weeks after suspension of roxadustat. There is accumulating evidence that roxadustat can cause CeH because of its similar structure to that of T3 [1-7]. In addition, Tanaka et al. recently reported that roxadustat-induced hypothyroidism is more likely to occur in elderly men [5]. Since the patient in our case was an elderly man, a greater focus on this possible adverse event may have been needed.

Importantly, our patient developed asymptomatic cerebral infarction three weeks after the change from roxadustat to daprodustat. Ultrasonography performed during hospital admission due to the onset of asymptomatic cerebral infarction revealed PFO and stable venous thrombosis in the lower limb, which is a risk factor for paradoxical cerebral embolism. In addition, the patient had diabetes, hypertension, and dyslipidemia for about 20 years, in addition to being elderly. Therefore, he had several risk factors for cardiovascular events. Whether the change from roxadustat to daprodustat provoked the onset of cerebral infarction in this case is not clear. However, after changing to daprodustat, elevation of Hb by about 2 g/dL (10.6 to 12.7 g/dL) occurred over a three-week period, despite daprodustat treatment at the minimum clinical dose. Therefore, the switch to daprodustat may exacerbate the onset of cerebral infarction, at least in part. Clear evidence is lacking for the effect on Hb of switching from one HIF-PH inhibitor to another, but our case suggests that caution is needed in making such a change, especially in patients at high risk for cardiovascular events.

## Conclusions

We encountered a patient who was treated with roxadustat for renal anemia and thereafter developed central hypothyroidism. After switching from roxadustat to daprodustat, the patient had an asymptomatic cerebral infarction. The association of the drug change with cerebral infarction was unclear, but the possible elevation of Hb after this change indicates the need for particular caution in this situation, especially in patients at high risk for cardiovascular events.

## References

[1] Haraguchi T, Hamamoto Y, Kuwata H (2023). Effect of roxadustat on thyroid function in patients with renal anemia. J Clin Endocrinol Metab.

[2] Kouki Y, Okada N, Saga K, Ozaki M, Saisyo A, Kitahara T (2023). Disproportionality analysis on hypothyroidism with roxadustat using the Japanese adverse drug event database. J Clin Pharmacol.

[3] Ichii M, Mori K, Miyaoka D (2021). Suppression of thyrotropin secretion during roxadustat treatment for renal anemia in a patient undergoing hemodialysis. BMC Nephrol.

[4] Tokuyama A, Kadoya H, Obata A, Obata T, Sasaki T, Kashihara N (2021). Roxadustat and thyroid-stimulating hormone suppression. Clin Kidney J.

[5] Tanaka H, Tani A, Onoda T, Ishii T (2024). Hypoxia-inducible factor prolyl hydroxylase inhibitors and hypothyroidism: an analysis of the Japanese pharmacovigilance database. In Vivo.

[6] Zheng X, Jin Y, Xu T, Xu H, Zhu S (2023). Thyroid function analysis after roxadustat or erythropoietin treatment in patients with renal anemia: a cohort study. Ren Fail.

[7] Yao B, Wei Y, Zhang S (2019). Revealing a mutant-induced receptor allosteric mechanism for the thyroid hormone resistance. iScience.

